# Human α-galactosidase A is stimulated by folic acid supplementation – possible implications in Fabry disease management

**DOI:** 10.1371/journal.pone.0351438

**Published:** 2026-06-10

**Authors:** Sabiha Khatoon, Mohammed A. Junaid

**Affiliations:** Department of Molecular Biology, New York State Institute for Basic Research in Developmental Disabilities, Staten Island, New York, United States of America; International University of Health and Welfare, School of Medicine, JAPAN

## Abstract

Fabry disease (FD) in an inherited lysosomal storage disorder with severe lifelong issues if not therapeutically managed. The disorder results from mutation in the gene *GLA* causing the deficiency of lysosomal enzyme α-galactosidase A (AGLA) leading to accumulation of specific glycosphingolipids in several organs. Individuals suffering from FD are treated with either an enzyme replacement therapy providing injected recombinant enzyme or small molecule chaperone therapy through a specific inhibitor, 1-deoxygalactonojiromycin (DGJ), which allows partial folding of functional enzyme that is retained as misfolded protein consequent to mutations. These therapies suggest that any incremental increase in AGLA activity will be of benefit to individuals with FD. This study is aimed at providing evidence that the synthetic B-complex vitamin, folic acid (FA) necessary in preventing neural tube defects in babies, can significantly stimulate the expression and enzyme activity in human lymphoblastoid cells as well as recombinant AGLA, in a concentration dependent manner. The extent of FA mediated AGLA stimulation was similar in cells from male or female subjects. In the recombinant enzyme, the stimulation follows mixed type with increase in Vmax but without any effect on Km. We did not find any evidence of cytosine methylation in one CpG island in the *GLA* gene. FA also stimulated and protected AGLA activity that was inhibited with specific inhibitor, DGJ. FA was able to further stimulate DGJ mediated restoration of AGLA activity in lymphoblastoid cells from individual with FD. Such FA mediated stimulation of AGLA activity may offer benefit to individuals with FD.

## Introduction

Fabry disease (FD) is an inherited disorder with severe lifelong manifestations due to accumulating glycosphingolipids in several tissues and organs [1]. FD is one of the lysosomal storage disorders marked by a severe deficiency of the lysosomal catabolic enzyme α-galactosidase A (AGLA) EC:3.2.1.22, arising from over 750 different mutations identified in the *GLA* gene so far [2,3]. This gene is located on the long arm of X-chromosome (chrX:101,393,273–101,408,012). Accumulation of specific lipids begins at birth in affected individuals, and continues throughout the lifespan, resulting in diverse signs and symptoms associated with the FD. Reduced life expectancy of males and females of 58.2 and 74.7 years respectively has been reported for people suffering from FD [4]. Earlier, a prevalence of FD was reported to be 1 in 40000–117000 worldwide, however, recent estimates based on largescale screening of neonates indicated prevalence of 1 in 3700 males in certain populations [5–7]. While genetic in nature, FD afflicts all racial and ethnic groups across the globe and is not concentrated in any specific community. Because *GLA* is located on X-chromosome and follows X-linked inheritance pattern, it often goes undetected in female population or is missed entirely [8]. There are two different types of FD recognized based upon the age of onset of the symptoms – a classic early onset displaying symptoms as early as two years of age, and a variant late onset with symptoms unnoticed sometimes until third decade of life [9,10]. A recent study has pointed out certain genetic variants not falling within this classification system and not displaying any blood accumulation of globotriaosylsphingosine [11].

*GLA* codes for the enzyme AGLA that functions as a homodimeric protein in lysosomes, where it catabolizes the hydrolysis of the terminal α-galactosyl residues from various glycosphingolipids [12]. Due to lack of AGLA activity in FD, specific glycosphingolipids, such as globotriaosylceramide (Gb3) accumulates in various cell types leading to clinical manifestation of the FD [13]. Primary symptoms of FD include renal insufficiency leading to kidney failure, cardiac involvement causing premature myocardial infarction and CNS pathology often leading to early stroke. Without proper treatment, invariably FD results in early death between the fourth and the fifth decade of an affected individual’s life [14].

Majority of *GLA* mutations are missense, affecting the structural integrity of the AGLA protein, and consequently the enzyme activity [15]. Residual enzyme activities with certain missense mutations are well correlated with the reduced severity of the clinical symptoms. To alleviate associated symptoms and delay disease progression, promising FDA approved treatments are available for FD patients depending on the type of mutation present in the *GLA*. Foremost being enzyme replacement therapy (ERT) that involves recombinant AGLA infused intravenously every two weeks to affected individuals [16]. This form of treatment is indicated for individuals with all types of mutations resulting in a lack of or severely diminished AGLA activity. Fabrazyme (agalsidase beta) and Elfabrio (pegunigalsidase alfa) are the two enzyme replacement medications available in the market. The other form of therapy is a small molecule oral chaperone therapy, that involves repairing a mutant AGLA protein that has misfolded because of missense mutations. A specific AGLA inhibitor, Migalastat also known as Galafold (1-deoxygalactonojiromycin) allows for a misfolded protein retained in the endoplasmic reticulum to undergo proper folding thereby regaining partial AGLA activity [17,18]. This type of treatment is restricted to those individuals who have a missense mutation leading to retained misfolded protein in the endoplasmic reticulum. Both types of treatment strategies suggest that any incremental increase in AGAL will substantially alter severity of disease progression allowing for improvement in the quality of life of affected individuals.

Aim of this study is to show evidence that synthetic folic acid (FA) supplementation substantially stimulates AGLA activity in human lymphoblastoid cells, as well as of pure recombinant human enzyme, in a concentration dependent manner. We further show that small molecule chaperone mediated rescue of AGLA in a patient’s cell line, is stimulated by FA, again in a concentration dependent manner. FA or vitamin B9, a water-soluble component of vitamin B complex, is important in nucleotide synthesis, DNA methylation and biological reactions of one-carbon metabolism, wherein its metabolites act as co-factor for several enzymes. It is routinely indicated in women of childbearing age to prevent occurrence of neural tube defects during gestational development.

## Materials and methods

4-methylumbelliferone (4-MU), 4-methylumbelliferyl-α-D-galactopyranoside (4-MU-α-Gal), FA, N-acetyl-D-galactosamine (NAG) and routine chemicals were purchased from Sigma-Aldrich (St. Louis, MO). RPMI 1640 media, α-galactosidase A rabbit recombinant monoclonal antibody (MA5–35650) and Falcon tissue culture dishes were purchased from ThermoFisher Scientific (Grand Island, NY). Fetal bovine serum, and heat inactivated, dialyzed fetal bovine serum were bought from Atlanta Biologicals (Flowery Branch, GA). Recombinant α-galactosidase A bearing human sequence representing residues 1–429 (Accession # P06280) with a C-terminal 6-His tag, > 95% purity was purchased from R&D Systems (Minneapolis, MN). Deoxygalactonojirimycin hydrochloride (DGJ) was purchased from Santa Cruz Biotechnology (Dallas, TX). RT-PCR primer pairs HP200154 for GLA (NM_000169) and HP200179 for HPRT1 (NM_000194) were purchased from Origene (Rockville, MD).

### Cell culture

Lymphoblastoid cells were obtained from the NIGMS Human Genetic Cell Repository at the Coriell Institute for Medical Research, Camden, NJ. Normal male and female lymphoblastoid cell lines GM7027 and GM7053, respectively, belonged to reference CEPH/UTAH Pedigree 1340. Cell line GM04391 also used in the current study was established from an individual diagnosed with FD. These three deidentified cells from the NIGMS Human Genetic Cell Repository at the Coriell Institute for Medical Research, Camden, NJ are publicly available to researchers to advance scientific research through Material Transfer Agreement. Written consents for use will be available with the repository. Authors have not obtained the original samples to establish cell lines.

The current study was conducted under the IRB protocol number NK12025−71, reviewed and approved by the Institutional Review Board of the Nathan Kline Institute for Psychiatric Research and Rockland Psychiatric Center, Orangeburg, NY.

Stock solution of sodium salt of FA was dissolved in water. It was diluted in either RPMI medium for cell exposure, or in citrate-phosphate buffer for recombinant enzyme assay. The maximum dilutions of FA we used in our study up to 500 ng/ml had no effect on fluorescence readout of 4MU. Fluorescent plate always had appropriate blanks; however, FA did not affect 4MU fluorescence.

These cell lines were grown in RPMI 1640 medium, supplemented with 15% fetal bovine serum, 2 mM glutamine, 1 mM sodium pyruvate and antibiotics (penicillin and streptomycin, 100 µg/ml each) as described earlier [19]. Prior to exposure to FA, cells were grown for 72 hours in RPMI 1640 FA free media containing 15% dialyzed and heat inactivated fetal bovine serum, 2 mM glutamine, 1 mM sodium pyruvate and antibiotics (penicillin and streptomycin, 100 µg/ml each). The same media (8 ml) was supplemented with FA at concentrations ranging from 0–500 ng/ml medium in 25 cm^2^ Nunc EasYFlask™ cell culture flasks at a density of 8 x 10^6^ cells. Cells were grown in a humidified incubator maintained at 37 °C in an atmosphere comprising of 5% CO_2_ in air. After three days of exposure to FA, cells were collected by centrifugation (800 x g for 5 min), rinsed twice with ice-cold normal saline, and stored frozen at −80 °C until further processed.

### Nucleic acids extraction, cDNA synthesis

DNA, RNA and proteins were extracted from frozen lymphoblastoid cells by Qiagen AllPrep DNA/RNA/Protein isolation kit according to manufacturer’s stated procedure. Amounts and purity of DNA and RNA eluted were determined by SmartDrop by Accuris Edison, NJ. RNA was stored at −80 °C, whereas DNA was stored at −20 °C until further used. cDNA synthesis was performed with 100 ng RNA using Qiagen first strand cDNA kit as described earlier [20].

### Quantitative PCR

Quantitative PCR was performed by using the BioRad CFX Opus 96 system (BioRad, Hercules, CA) in combination with the RT2 SYBRGreen PCR Master Mix (Qiagen) as described earlier [21]. Each reaction was run in duplicate and repeated three times from different batches of cDNA. HPRT1 was used as endogenous control for amplification. Relative gene expression was calculated by using the Pfaffl method of Ct values [22].

### Cytosine methylation

Targeted region of interest (ROI) bisulfite sequencing was performed for a 573 bp fragment of *GLA* gene comprising of nucleotides chrX:101407790–101408363 at Zymo Research Corporation (Irvine, CA). This fragment comprises 34 CpG sites within a single CpG island.

### Cell free extracts

Cell pellets were resuspended in 200 µl of 25 mM citrate-phosphate buffer pH 4.6 and extracts were prepared with Fisherbrand Sonifier FB505 (ThermoFisher Scientific) equipped with cup horn as described earlier [23]). Clear supernatant obtained after centrifugation at 14,500 rpm for 10 minutes was stored frozen at −20 °C until used. Protein concentrations were measured by the modified Folin phenol method using BSA as the standard [24].

### AGLA activity

AGLA was measured in 96-well plates as described [25,26]. Briefly, 50 µl of extract in 25 mM citrate-phosphate buffer pH 4.6 was incubated with 50 µl of the substrate (2 mM 4-MU- α-Gal dissolved in citrate-phosphate buffer, pH 4.6) for up to 3 hours at 37 °C under shaking conditions. Reaction was inhibited by mixing with 100 µl of 0.1 M glycine-carbonate buffer, pH 11. To ensure measurement of specific AGLA activity in cell extracts, the assay was also determined in the presence of NAG as described [27]. Briefly, extract was incubated with 6 mM 4-MU-α-Gal and 90 mM NAG in 100 µl of 0.1M citrate-phosphate buffer pH 4.5 for up to 30 minutes. Reaction was terminated by mixing 100 µl of 0.2 M glycine-carbonate buffer, pH 10.5. Product formation was measured in Spectramax M3 plate reader (Molecular Devices, San Jose, CA) using excitation and emission wavelengths 365 nm and 450 nm, respectively. Florescence readings were converted to molarities using standard curve generated with defined concentrations of 4-MU in same chemical composition. A master mix with cell free extract was prepared when incubations were made with the FA.

### Western blotting

Western blot analysis was done as described earlier [19]. Non-specific sites on the membranes were blocked for two hours with 5% nonfat dry milk in TBST, incubated with primary antibody (1:250) overnight at 4 °C, then incubated with horse radish peroxidase–conjugated secondary antibodies (1:2,000) at room temperature for two hours, and developed by using the SuperSignal West Pico chemiluminescent substrate (ThermoFisher, Rockford, IL). Images were captured on an iBright FL1500 Imaging System (ThermoFisher, Rockford, IL).

### Statistics

Each experiment was performed in triplicates in three independent experiments. Values are presented as means ± SEM. Significance of the difference of AGLA activity in the presence or absence of FA at each concentration was determined by upaired t-tests; significance of overall effects was determined by ANOVAs. The increase in AGLA activity with logarithmically increasing FA concentration was tested with an OLS regression. A significance criteria of *P* < 0.05 was applied throughout.

## Results

**Fig 1:**
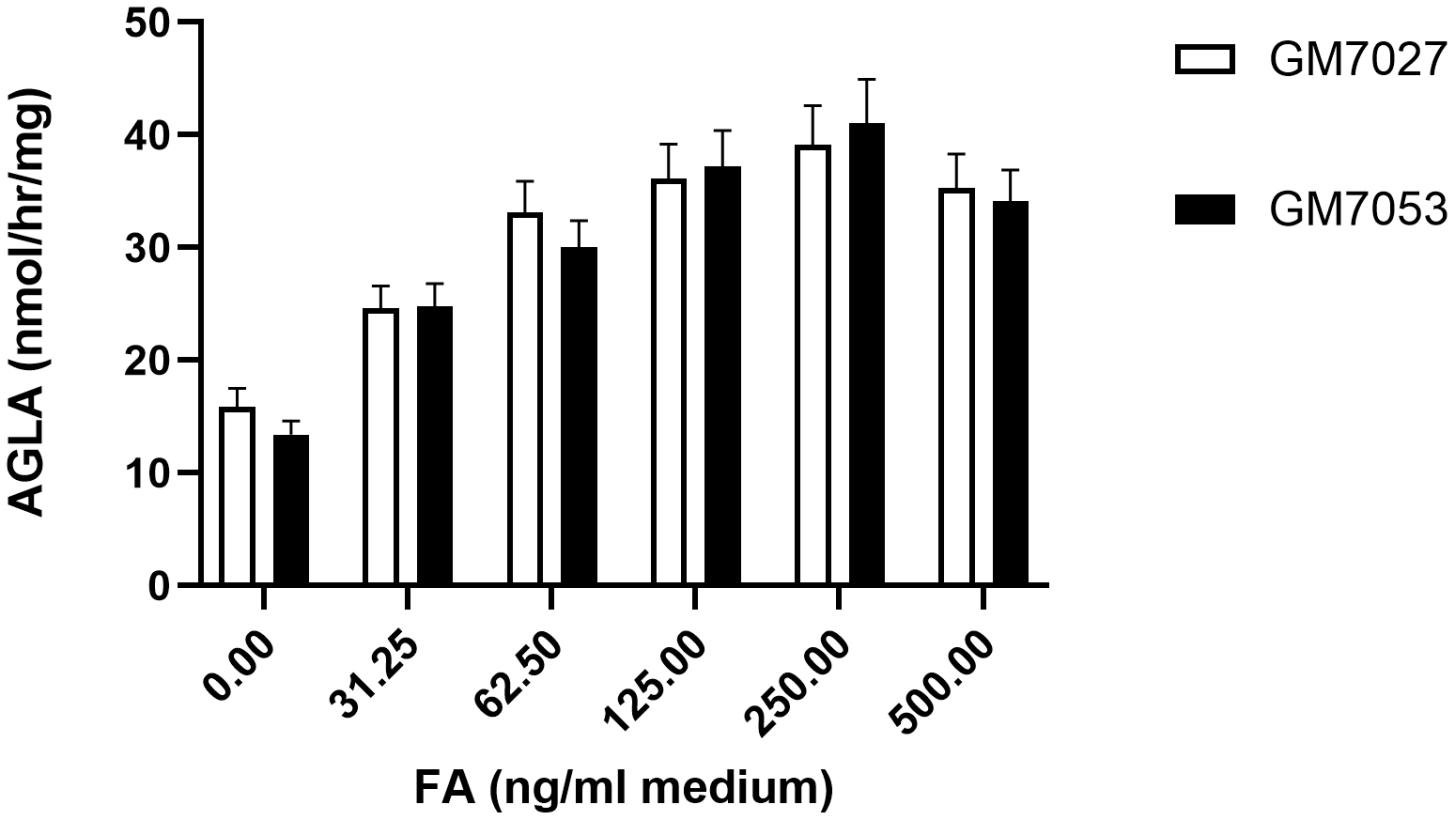
AGLA activity in lymphoblastoid cells. Cell lines GM7027 (male) and GM7053 (female) were grown in FA free media for three days prior to incubating with the indicated FA concentrations in RPMI 1640 medium. After three days, cells were collected by centrifugation, rinsed with normal saline, and AGLA activity determined in presence of NAG. Values are means of three independent experiments and bars show SEM. AGLA values at each FA concentration was significantly different (*P* < 0.0001) from that at zero concentration.

When lymphoblastoid cells in culture conditions were exposed to FA supplementation, it caused significant stimulation of the *GLA* transcript, protein, and AGLA enzyme activity, in a concentration dependent manner. Enzyme activity measurements in cell free extracts displayed that AGLA activity in lymphoblastoid cells was increased significantly with as low FA concentration as 31.25 ng/ml medium (Fig 1). Increased AGLA enzyme activity was measurable with the highest FA concentration tested at 250 ng/ml medium. At higher concentrations FA-mediated stimulation begins to diminish, and in fact it appears to inhibit AGLA activity. The stimulation of AGLA activity by FA was similar in extent regardless of whether the cell line was established from a male or a female subject (p = 0.67).

The FA mediated increase in AGLA activity seen in lymphoblastoid cells appears highly specific, as it was determined in presence of NAG, and since enzyme activities of several other lysosomal enzymes tested remain unchanged upon incubation with similar FA concentrations. We tested enzyme activities of aryl sulfatase A (EC:3.1.6.8), aryl sulfatase B (EC:3.1.6.12), α-L-fucosidase (EC:3.2.1.51), β-D-galactosidase (EC:3.2.1.23), α-D-glucosidase (EC:3.2.1.20), β-glucoronidase (EC:3.2.1.31), α-D-mannosidase (EC:3.2.1.24), and tripeptidyl peptidase (EC: 3.4.14.9) in lymphoblastoid cells upon FA supplementation for 3-days, and found that at all concentrations these enzyme activities were unaffected (Supplementary Information S1 Table). The stimulation determined in the presence of NAG indicates that the effect is specific to AGLA and not to competing α-N-acetylgalactosaminidase (EC:3.2.1.49), which also cleaves the substrate used. NAG inclusion in assays with recombinant AGLA showed no effect on the stimulation as expected.

**Fig 2:**
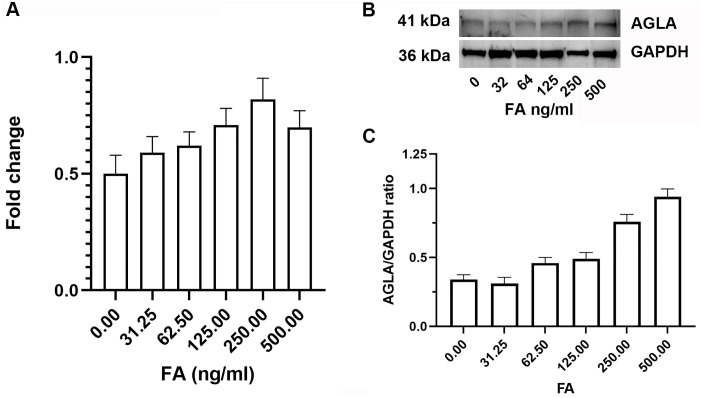
FA supplementation stimulates AGLA expression in lymphoblastoid cells at transcript and protein levels. Cells were grown in FA free medium for three days prior to incubating with the indicated FA concentrations in RPMI 1640 medium. After three days, cells were collected by centrifugation, rinsed with normal saline, and RNA or proteins were prepared. **(A)** Transcript levels were measured by RT-PCR using primer pair HP200154 for *GLA* and HP200179 for *HPRT1*. Values are mean of three independent experiments and bars show the SEM. Differences among concentrations were significant (F (5,12) = 3.85, p < 0.026). **(B)** Western blot analysis of lymphoblastoid cells exposed to FA concentrations, (25 µg protein each) were probed with an anti-AGLA rabbit monoclonal antibody. Picture of a representative blot. **(C)** Densitometric measurement of blots using Image J, and expression of ratio with the housekeeping gene GAPDH. Differences among concentrations were extremely significant (F (5,12) = 19.81, *p* < 0.0001).

Quantitative RT-PCR data using validated primer set confirmed increased expression of *GLA* transcript in lymphoblastoid cells supplemented with FA (Fig 2A). The primer set utilized forward 5’GCAACCTTGACTGCCAGGAAGA3’ and reverse 5’ CTCATAACCTGCATCCTTCCAGC 3’ primers, respectively, which amplifies a ~ 107 bp fragment about 150 bp into the ORF. This primer pair amplifies isoforms 1, 3, 4 and 5. In our amplifications, a single peak was displayed in melting curve. Transcript levels at zero FA were compared with those at indicated concentrations. AGLA transcript values differed significantly by FA concentration (F (5,30) = 51.46 *p* < 0.001). At FA concentrations below 31.25 ng/ml AGLA expression was also higher but the effect was not statistically significant. The increase in AGLA expression with increasing FA concentration was strongly significant (*p* < 0.001). Western blot analysis using an anti-AGLA rabbit monoclonal antibody displayed that AGLA protein expression increased in lymphoid cells with increasing FA concentration in the medium. There is a basal level of expression of AGLA in the absence of FA, and thereafter the AGLA expression increased in a concentration dependent manner with increasing FA concentration (Fig 2B). The lowest concentration we have tested that showed increased expression is 31.25 ng FA/ml of RPMI 1640 medium. We used GAPDH for housekeeping gene, as expression of this gene remained unchanged with increasing FA concentrations [19].

**Fig 3:**
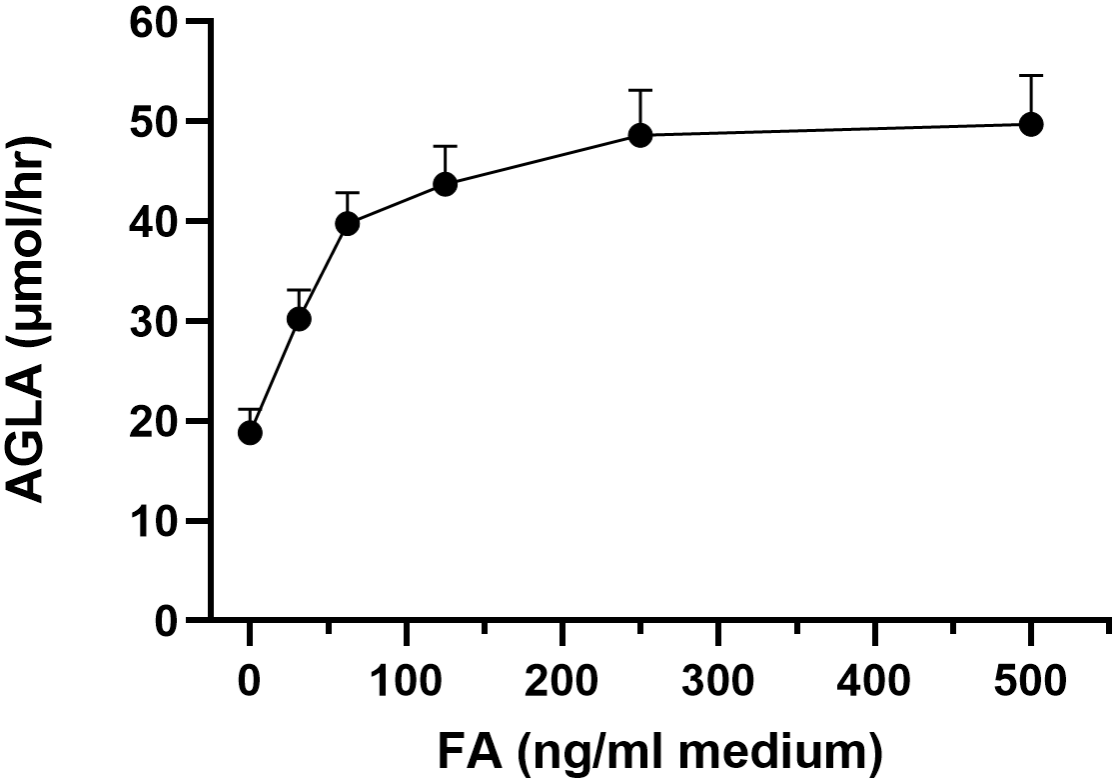
Stimulation of recombinant AGLA by FA. Recombinant AGLA (Accession # P06280) activity was determined in the presence of indicated FA concentration using 4-MU-α-Gal as the substrate. Release of 4-MU was determined fluorometrically. Values represent means with bars showing SEM for three independent measurements. AGLA activity was significantly different (*P* < 0.05) at all concentrations when measured against zero FA.

**Fig 4:**
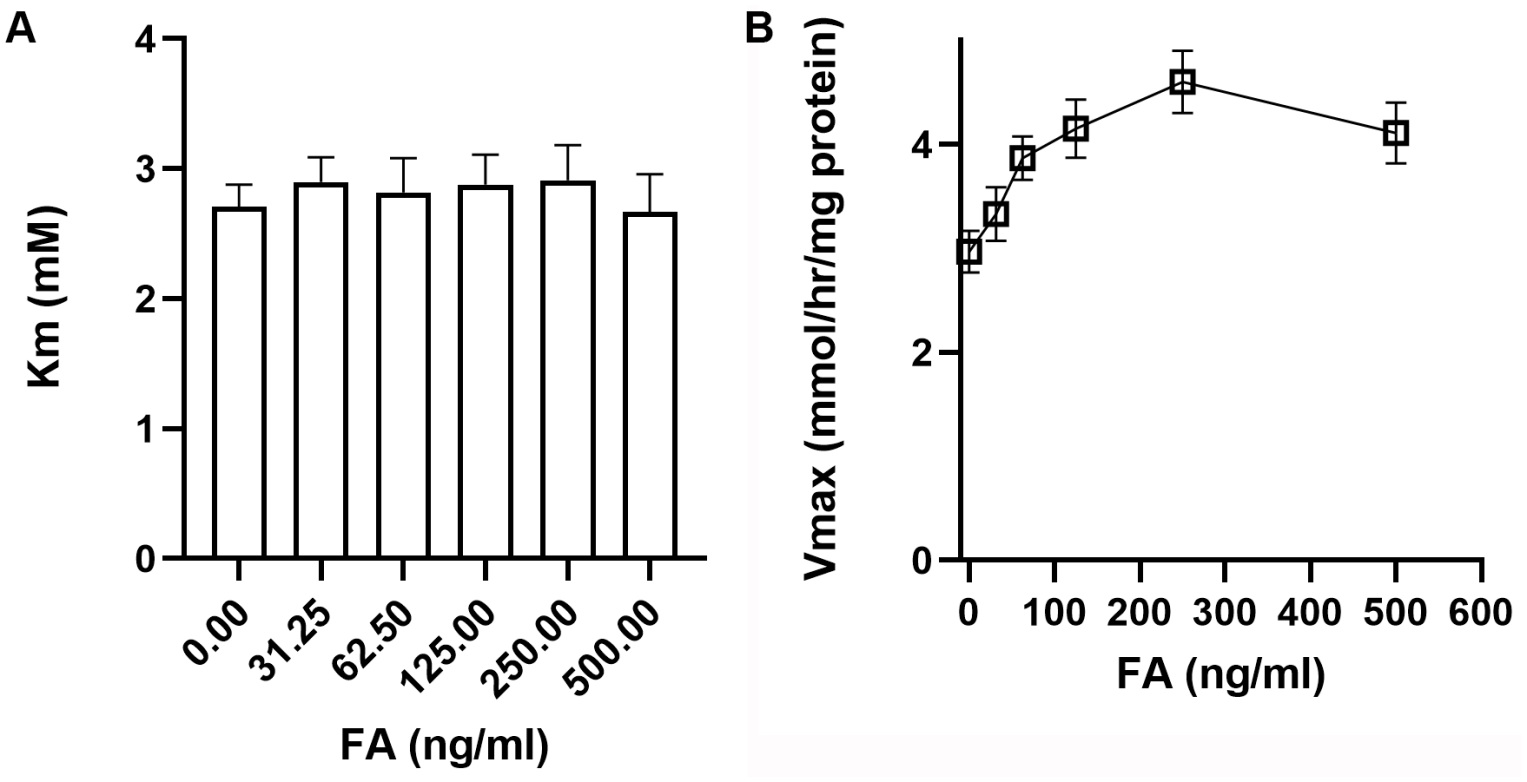
Kinetic properties of recombinant AGLA in the presence of FA. AGLA activity was determined at various 4-MU-α-Gal concentrations at indicated FA concentration. Km (A) and Vmax (B) were determined from double reciprocal Lineweaver-Burk plots. Values are means and bars show SEM of three independent measurements.

We tested whether enzyme activity of the purified AGLA protein is also stimulated with FA incubation. We used the recombinant human AGLA protein with a C-terminal 6-His tag, expressed in CHO cell line (R&D systems, 6146-GH) as the pure enzyme. As shown in Fig 3, AGLA activity is stimulated by *in vitro* incubation with FA, again in a concentration dependent manner showing a similar trend as lymphoblastoid cells with respect to FA concentrations. The difference in AGLA expression by FA concentration was quite significant (F (5,12) = 20.58, *p* < 0.0001). AGLA activity determined in the *p*resence of NAG failed to show any inhibition even at the lowest FA concentration indicating that NAG, as expected, has no effect on the pure AGLA activity. Kinetic parameters evaluated of the AGLA activity at various concentrations of FA demonstrated that the stimulation of the enzyme activity is of mixed type with increasing Vmax but unchanged Km (Fig 4). Km was not statistically significant between zero and various FA concentrations (F (5,12) = 0.41, *p* = 0.85), whereas Vmax at FA higher than 62 ng/ml was significantly different (F (5,12) = 9.72, *p* < 0.0007). Vmax at 31.25 ng/ml FA showed upward trend but was not statistically significant.

**Fig 5:**
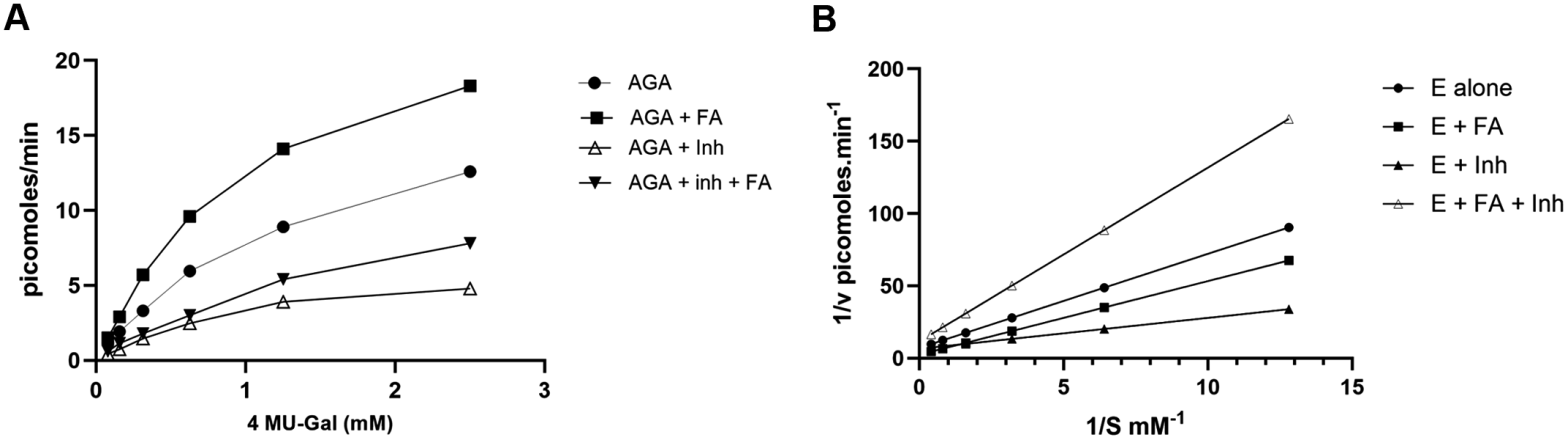
FA reverses DGJ induced inhibition of recombinant AGLA activity. Recombinant AGLA was incubated with or without the specific inhibitor DGJ, either in the presence or absence of FA at varying concentrations of the substrate, 4MU-Gal **(A)**. Fluorescence was measured after terminating the reaction with the stop solution. **(B)** Lineweaver-Burk plot of the data to determine type of enzyme mechanism.

We then tested whether recombinant AGLA activity inhibited by specific inhibitor, 1-deoxygalactonojirimycin (DGJ) (Migalastat or Galafold) is restored by FA incubation. As shown in Fig 5, FA incubation of AGLA activity inhibited by DGJ is significantly restored by FA incubation by over 50% at increasing FA concentrations. The order of preincubation of AGLA with either FA or the inhibitor displayed interesting results. When AGLA was preincubated with the inhibitor followed by addition of FA, this restored the AGLA activity by about 50%. On the other hand, preincubation of AGLA with FA for 15 minutes on ice, followed by addition of the inhibitor offered additional 15% protection of AGLA activity from being inhibited by the inhibitor (Table 1).

**Table 1:** FA preincubation with AGLA is beneficial.

Condition	pmol. Min^-1^
Enzyme alone	9.42 ± 0.4
Enzyme + DGJ (7 µM)	2.47 ± 0.2*
Enzyme + DGJ + FA	6.06 ± 0.5*
Enzyme + FA + DGJ	6.96 ± 0.5*

Recombinant AGLA was incubated with or without DGJ at 7 µM. AGLA activity was measured by incubating with FA that was added either along with DGJ, or 15 minutes prior to adding DGJ on ice. Values represent mean ± SEM of three independent experiments and * denotes significantly different from activity without DGJ (*P* < 0.05).

**Fig 6:**
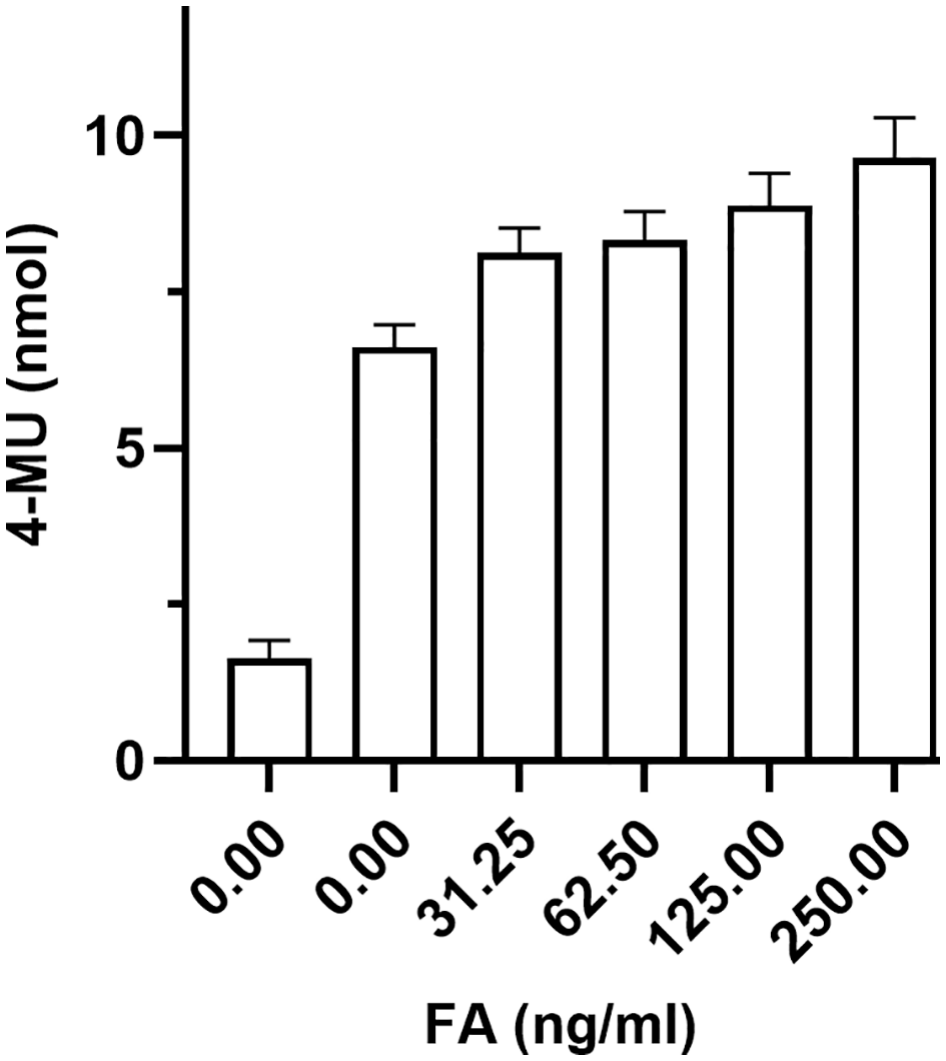
FA stimulates DGJ mediated rescue of AGLA activity in lymphoblastoid cells from a FD individual. AGLA activity in the cell line GM04391 established from a FD individual was measured without or with DGJ (7 µM), at indicated concentrations of FA in the media. Values represent mean ± SEM of three independent experiments.

We also tested whether AGLA activity restored by small molecule chaperone DGJ therapy compound shows any effect upon incubation with FA. The AGLA activity in lymphoblastoid cells from an individual with FD was low (Fig 6). After incubation with 7 µM concentration of DGJ, the AGLA activity was increased by > 4-folds compared to cells incubated without DGJ. This AGLA activity, restored by DGJ in the lymphoblastoid cells, was further stimulated by incubation with FA again in a concentration dependent manner, with the FA even at 31.25 ng/ml level stimulating AGLA activity by a further 18% (a 1.2-fold increase over DGJ restored level). The restored AGLA activity continues to increase further with increasing FA concentration of 250 ng/ml. The overall effect was strongly significant (F (5,12) = 74.05, *p* < 0.0001).

We performed targeted methylation sequence of one CpG island (573 bp) in *GLA* gene near the promoter region to see if FA mediated differential methylation of cytosine residues. The results indicated that none of the 34 cytosines in the dinucleotide CpG sites analyzed within this 573 bp fragment had higher methylation rate compared to cells incubated without FA (Supplementary Information, S1 Fig and S2 Table).

Together, the data from this study clearly demonstrates that FA supplementation increases AGLA mRNA and protein expression in the lymphoblastoid cells and further, the purified enzyme activity is also stimulated by FA incubation, in concentration dependent manner. Additionally, we provide convincing proof that AGLA activity restored by small molecule chaperone DGJ therapy is further augmented by incubation with increasing FA supplementation.

## Discussion

FD is a debilitating inherited disability afflicting people of all ethnicities around the globe. The introduction of promising therapies in the form of enzyme replacement and chaperone therapies have alleviated suffering individuals’ difficulties and contributed significantly to improved lifestyles [28]. Successes of both therapies demonstrated that any incremental increase in the AGLA activity in affected individuals is of paramount importance in lessening the symptoms severity. Our current results indicated that both types of therapies may greatly benefit if FA supplementation is simultaneously included with these treatment modalities.

While screening beneficial and potentially detrimental epigenetic effects of the vitamin FA, we observed potential beneficial effects of FA supplementation through substantially augmenting the AGLA activity in human lymphoblastoid cells and the pure recombinant enzyme. Mandatory synthetic FA supplementations of breakfast cereals and grains was instituted to prevent neural tube defects (NTDs) in newborns [29]. This single dietary modification is credited with over 70% reduction in incidences of NTDs. Thus, there is widespread use of FA supplementation in the general population. The exact molecular mechanism involved in FA preventing NTDs are unknown, however, FA is cofactor in enzymes that participate in one-carbon metabolism, and it is expected that combinations of such metabolic activities apart from stimulated nucleic acid synthesis may be responsible.

Our current work clearly demonstrates that FA supplementation has hitherto unknown beneficial impact by stimulating AGLA through two separate but interconnected mechanisms involving production of AGLA at the gene expression level, and a possible direct interaction with the mature enzyme. First, FA supplementation of lymphoblastoid cells in culture stimulated AGLA mRNA and protein expression, which is demonstrated by increasing transcript and protein levels. This is happening at the level of augmented gene expression, which may be consequent to epigenetic effects of FA. However, we did not find any of the cytosine residues that we tested within a single CpG island showing any differential methylation status under FA supplementation to support that this particular CpG island participates in any direct epigenetic effect. These 34 CpG sites we tested in one CpG island are in the vicinity of the gene promoter. This suggests that FA is not exerting any direct epigenetic effect on the one CpG island tested, which argues that additional CpG sites may be present in the *GLA* gene, or FA may promote augmented AGLA expression through another yet unidentified gene that may regulate the *GLA* gene expression. This remains to be established.

Second, *in vitro* FA incubation also increases AGLA activity in a concentration dependent manner, which is reflected by stimulation of pure recombinant AGLA protein. Kinetic measurement indicated increase in Vmax but not Km of the enzyme. The binding sites for FA and substrates are expected to be different since enzyme activity stimulation was found to be of mixed type and without any impact on the Km. Thus, FA is also able to stimulate the AGLA enzyme activity as a direct modulator by interacting with a site not involving active site residues. In the past, glucose, melibiose and raffinose sugars were shown to stimulate AGLA activity, while trehalose treatment induced a marginal increase at much higher concentrations [30]. Thus, FA seems to be a more potent stimulator of AGLA activity both under *in vivo* and *in vitro* conditions.

Irrespective of the gender, the increase in AGLA activity was comparable in both male and female lymphoblastoid cell lines with all FA concentrations tested. This is important since *GLA* gene is localized on X-chromosome, and women do exhibit X-linked inheritance issues for *GLA* expression due to which they often experience delayed diagnosis for FD [31].

The FA mediated stimulation of AGLA was found to be highly specific for this lysosomal enzyme, since no such stimulation was observed with several other lysosomal enzymes tested. Stimulation in the presence of NAG also rules out effect of α-N-acetylgalactosaminidase (EC:3.2.1.49), the enzyme that also utilizes the substrate used. Also, of importance is β-galactosidase 1(EC:3.2.1.23) that acts on substrate showing only a change in glycosidic linkage orientation in comparison to AGLA [32]. Such specificity is expected, since both AGLA and beta-galactosidase 1 are separate proteins encoded by different genes. Thus, structurally, there may be motifs in AGLA that are amenable to binding FA and consequently stimulating the enzyme activity, and such motifs may be lacking in other lysosomal enzymes we tested. In a similar manner, DGJ specifically inhibits the AGLA activity while no such inhibition is observed with beta-galactosidase 1 [27]. Earlier reports had found that AGLA activity is stimulated by ammonium chloride possibly through change in microenvironment at the lysosomal level [33]. Sphingolipid activator protein B (saposin B) stimulates the degradation of the accumulated material globotriaosylceramide [34], presumably through micellar structure formation and recognition of the same by AGLA [35]. Quinovose (6-deoxy-D-glucose) was specifically shown to stimulate the induction of an extracellular AGLA from *Talaromyces flavus* [36]. Trehalose, a disaccharide made of two glucose units stimulates several lysosomal hydrolases including the AGLA through autophagy mediated mechanism [30]. Thus, our research has found a potent small molecule at low concentration, which is routinely consumed as a supplement, which can substantially stimulate AGLA activity.

The exact mechanism of how FA is stimulating pure AGLA is presently unclear. Whether FA is a cofactor for AGLA is unknown at this time, however, it will be worth investigating FA binding to AGLA and clarify such a role. FA does participate as cofactor in enzymes involved in one-carbon metabolism. It is possible that FA binds to AGLA and stabilizes the enzyme, which is reflected from added stimulation of AGLA upon prior FA incubation before inhibitor is added. Lowest concentration of FA shown in our experiments to increase the level of AGLA both at transcription as well as *in vitro* activity level is lower than what has been reported in the blood FA concentrations in the population following FA supplementation [37].

Our research also provides direct proof that FA can substantially stimulate small molecule chaperone DGJ mediated refolding of AGLA in both, the recombinant AGLA as well as in a cell line from a FD affected individual. DGJ stabilizes the misfolded protein and helps it refold in proper conformation resulting in partial restoration of enzyme function [28]. FA incubation further stimulates the refolded enzyme thereby augmenting AGLA enzymatic activity. According to the NIGMS Human Genetic Cell Repository at the Coriell Institute for Medical Research, Camden, NJ, the lymphoblastoid cell line tested was from an individual with confirmed FD diagnosis. This individual is a male and was 41 years old at the time of establishing the cell line. Genetic analysis revealed him to be hemizygous for an A > G transition in exon 5 of the *GLA* gene (codon AAT > AGT) resulting in Asn215Ser substitution in the protein. The individual had residual AGLA activity in fibroblasts and WBC and displayed mild clinical manifestations [38]. Because FA is stimulating the pure AGLA activity, its inclusion in small molecule chaperone DGJ-based therapy may also nullify any inhibition the AGLA may encounter by the presence of DGJ.

Our study is limited to a single cell line from an individual with FD. It needs to be replicated in FD individuals with other types of missense mutations with classic phenotypes. Another limitation is we also did not measure any disease relevant substrate in the cell lines in response to FA supplementation, that may provide conclusive evidence of the supplementation benefit.

Stimulation of AGLA by FA supplementation may offer benefits to individuals with FD on either ERT or chaperone therapy. Our current research has shown the potential that FD individuals with residual AGLA activity may be able to augment the enzyme activity further through FA supplementation with likely alleviation in the symptomology. Obviously this needs to be confirmed through clinical trials in individuals with FD. As a preliminary proof of concept, we have shown that FA supplementation further augments AGLA activity restored by the chaperone DGJ in lymphoblastoid cells from FD individual, and in pure recombinant enzyme.

## Supporting information

S1 Table Lysosomal enzymes tested in the current study with increasing FA concentration. Enzyme activities were tested by methods described in the reference quoted.
(PDF)

S2 Table CpG methylation status around *GLA* promoter region. GLA CpG island unique sequence methylation status of the 34 CG sites. The ratio of all these sites is below zero, indicating lack of methylation at any of the sites.
(XLSX)

S3 Table Data files. Data used in the current study for figures generated.
(XLSX)

S1 Fig *GLA* – CpG island. *GLA* CpG island unique sequence; Dark Gray shade is sequencing amplicon, Light Gray shade is region of interest; CG is CG of interest.
(PDF)

S1 Raw Images Original images of Western blot for AGLA staining used in Fig 2B upper and lower panels. (PDF)
