# Supplementary material for: Human α-galactosidase A is stimulated by folic acid supplementation – possible implications in Fabry disease management
Source: PLoS One. 2026 Jun 10;21(6):e0351438. doi: 10.1371/journal.pone.0351438 (PMC13252739; doi:10.1371/journal.pone.0351438)
Supplement: S1 Table — Enzyme activities were tested by methods described in the reference quoted. (PDF) [file pone.0351438.s001.pdf]

Table S1: List of lysosomal enzymes and their activities that were tested for effect of FA supplementation in GM 7053. Activities of these enzymes tested according to published articles remained unchanged with increasing FA concentrations.

Enzyme	Enzyme Activities (nmol/hr/mg protein)						Assay Reference
	FA concentrations (ng/ml)						
	0	31.25	62.5	125	250	500	
Aryl sulfatase A (EC:3.1.6.8)	26.02	25.9	27.0	26.6	25.4	26.5	Beratis NG, Danesino C, Hirschhorn K. Detection of homozygotes and heterozygotes for metachromatic leukodystrophy in lymphoid cell lines and peripheral leukocytes. Ann Hum Genet. 1975: 38(4):485-493. doi: 10.1111/j.1469-1809.1975.tb00638.x. PMID: 1238048.
Aryl sulfatase B (EC:3.1.6.12)	120	132	127	136	126	131	Beratis N, Turner B, Weiss R, Hirschhorn K. Arylsulfatase B Deficiency in Maroteaux-Lamy Syndrome: Cellular Studies and Carrier Identification. Pediatr Res. 1975; 9: 475–480. https://doi.org/10.1203/00006450-197505000-00003. PMID: 806052.
α-L-fucosidase (EC:3.2.1.51)	148	131	130	170	141	161	Fucosidosis: detection of the carrier state in peripheral blood leukocytes. Beratis NG, Turner BM, Hirschhorn K. J Pediatr. 1975; 87(6 Pt 2):1193-1198. doi: 10.1016/s0022-3476(75)80135-1. PMID: 1185419
β-D-galactosidase (EC:3.2.1.23)	68	76	79	71	69	75	Ho MW, Seck J, Schmidt D, Veath ML, Johnson W, Brady RO, O'Brien JS. Adult Gaucher's disease: kindred studies and demonstration of a deficiency of acid beta-glucosidase in cultured fibroblasts. Am J Hum Genet. 1972; 24(1):37-45. PMID: 5012691.
β-glucoronidase (EC:3.2.1.31)	250	275	263	272	266	271	Glaser JH, Sly WS. Beta-glucuronidase deficiency mucopolysaccharidosis: methods for enzymatic diagnosis. J Lab Clin Med. 1973; 82(6):969-77. PMID: 4202279.
α-D-glucosidase (EC:3.2.1.20)	35	28	32	34	33	34	Beratis NG, LaBadie GU, Hirschhorn K. Characterization of the molecular defect in infantile and adult acid alpha-glucosidase deficiency fibroblasts. J Clin Invest. 1978; 62(6):1264-74. doi: 10.1172/JCI109247. PMID: 34626.
α-D-mannosidase (EC:3.2.1.24)	0.21	0.21	0.22	0.20	0.21	0.20	Prence EM, Natowicz MR. Diagnosis of alpha-mannosidosis by measuring alpha-mannosidase in plasma. Clin Chem. 1992; 38(4):501-503. PMID: 1568314.
Tripeptidyl peptidase I (EC:3.4.14.9)	742	732	729	737	741	734	Junaid MA, Sklower Brooks S, Wisniewski KE, Pullarkat RK. A novel assay for lysosomal pepstatin-insensitive proteinase and its application for the diagnosis of late-infantile neuronal ceroid lipofuscinosis. Clin Chim Acta. 1999; 281(1-2):169-176. doi: 10.1016/s0301-2115(98)00333-9. PMID: 10217638.
