# Supplementary material for: Human α-galactosidase A is stimulated by folic acid supplementation – possible implications in Fabry disease management
Source: PLoS One. 2026 Jun 10;21(6):e0351438. doi: 10.1371/journal.pone.0351438 (PMC13252739; doi:10.1371/journal.pone.0351438)
Supplement: S1 Fig — GLA CpG island unique sequence; Dark Gray shade is sequencing amplicon, Light Gray shade is region of interest; CG is CG of interest. (PDF) [file pone.0351438.s004.pdf]

>hg38_dna range=chrX:101407790-101408363

ATCATCACCACCCCTGGGTCCCCAGTTCCCACCCACACACCAACCTCTAACGATACCGGGTAATTTTCCTCCTTC
TTCCCTCAAACGGCTATAGCGAGACGGTAGACGACGACCAGAACTACTTCTGCTCACGTAAGCGAGTAATCACGT
GAGCGCCTACGTCATGTGAGATCTCGGTCACGTGAGCAACTCTCGGCTTAAACTCGGGATCACTAAGGTGCCGCA
CTTCCTTCTGGTATGGAAATAGGGCGGGTCAATATCAAGAAAGGAAGAGGGTGATTGGTTAGCGGAACGTCTTAC
GTGACTGATTATTGGTCTACCTCTGGGGATAACCGTCCCAGTTGCCAGAGAAACAATAACGTCATTATTTAATAA
GTCATCGGTGATTGGTCCGCCCCTGAGGTTAATCTTAAAAGCCCAGGTTACCCGCGGAAATTTATGCTGTCCGGT
CACCGTGACAATGCAGCTGAGGAACCCAGAACTACATCTGGGCTGCGCGCTTGCGCTTCGCTTCCTGGCCCTCGT
TTCCTGGGACATCCCTGGGGCTAGAGCACTGGACAATGGATTGGCAAGG
