# Supplementary material for: Human α-galactosidase A is stimulated by folic acid supplementation – possible implications in Fabry disease management
Source: PLoS One. 2026 Jun 10;21(6):e0351438. doi: 10.1371/journal.pone.0351438 (PMC13252739; doi:10.1371/journal.pone.0351438)
Supplement: S1 Raw Images — (PDF) [file pone.0351438.s005.pdf]

Lanes X 2 3 4 5 6 7 X Std kD

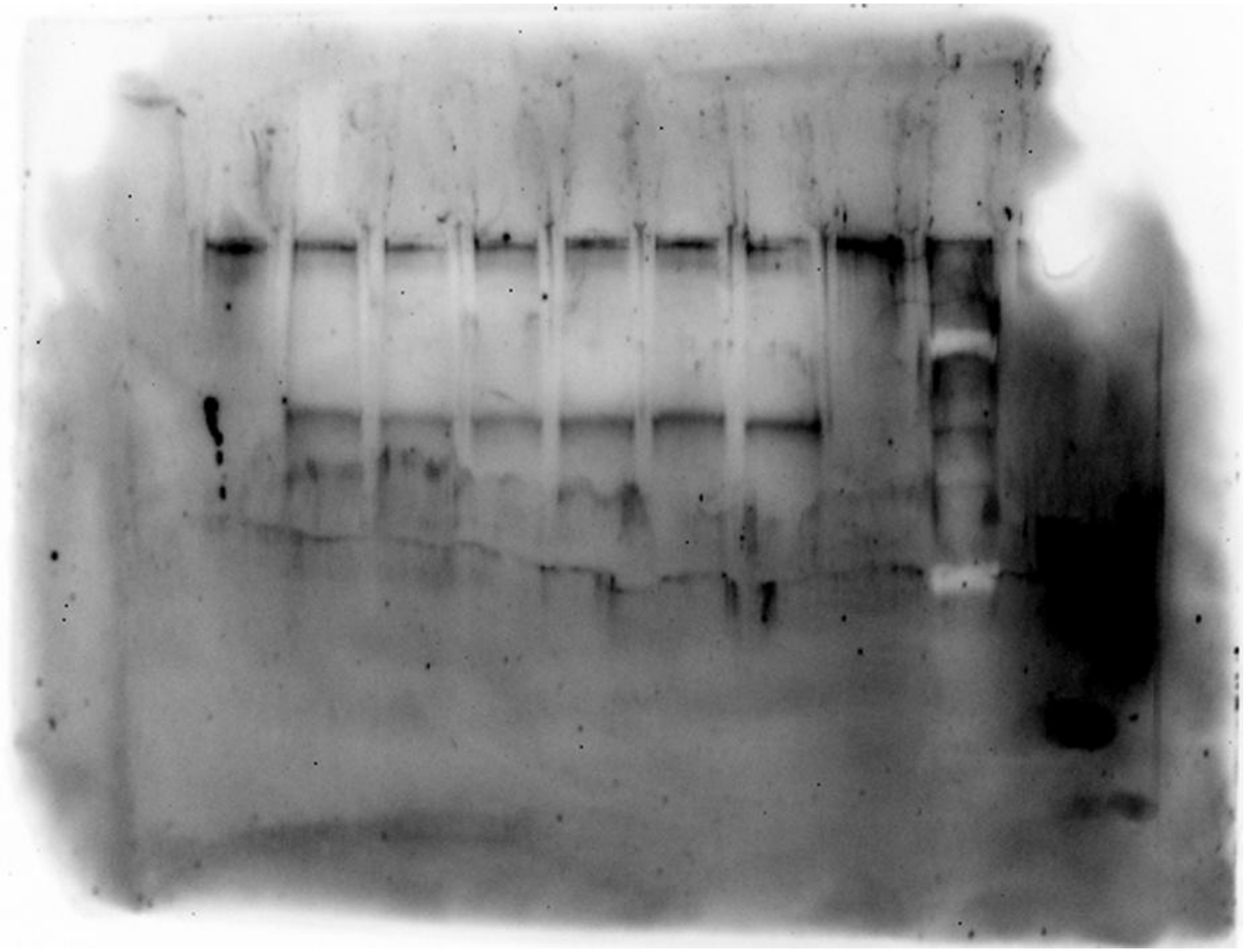

250
150
75
50
37
25
20

Original, uncropped and unadjusted blot of anti-AGLA staining used in Figure 2B upper panel of the manuscript. Lanes 2-7 from left are cell extracts with increasing FA supplementation from 31.25, 62.5, 125, 250 and 500 ng/ml media. Images were captured on an iBright FL1500 Imaging System (ThermoFisher, Rockford, IL) in using ChemiBlot SmartCapture.

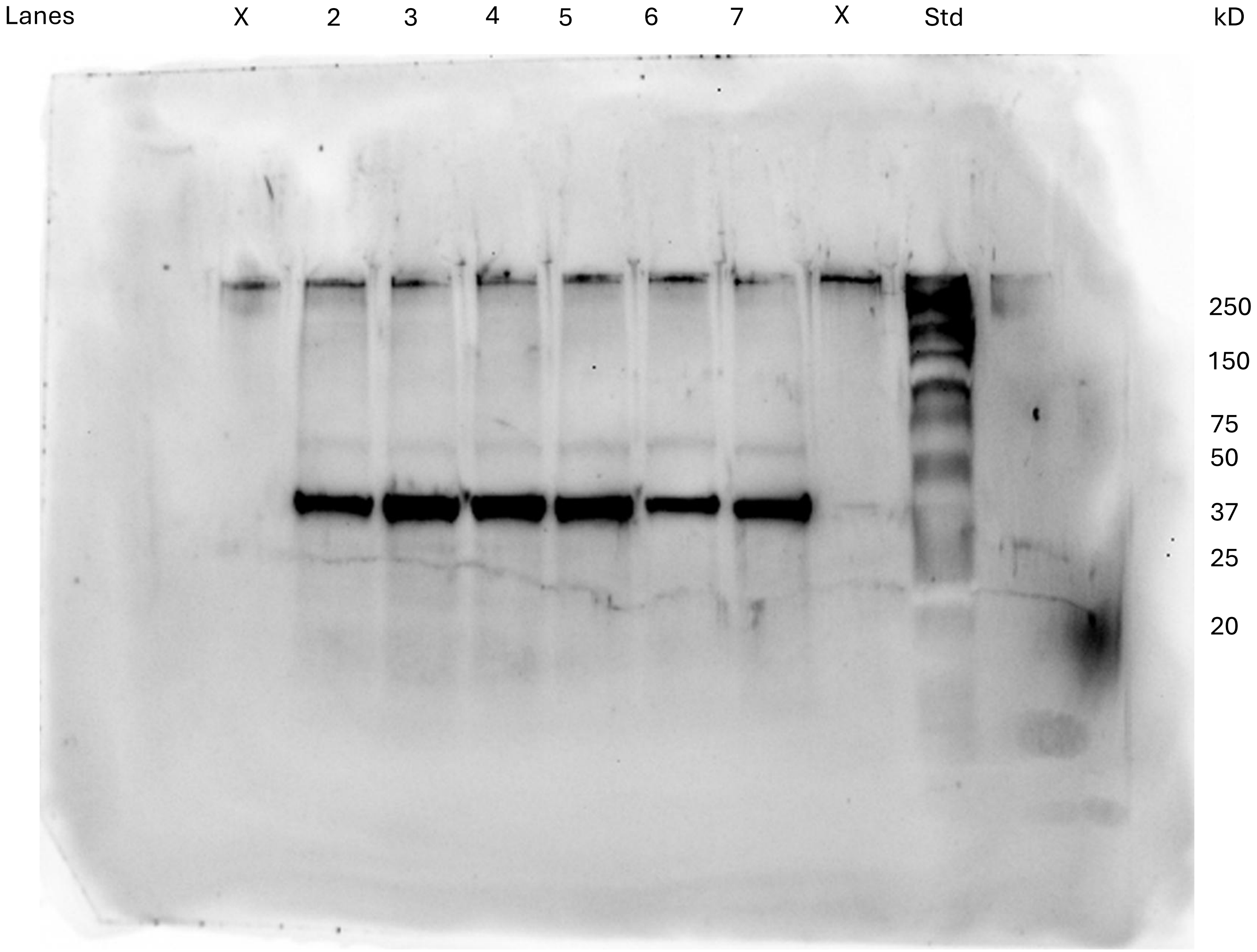


Original, uncropped and unadjusted blot of anti-GAPDH staining used in Figure 2B lower panel of the manuscript. Lanes 2-7 from left are cell extracts with increasing FA supplementation from 31.25, 62.5, 125, 250 and 500 ng/ml media. Images were captured on an iBright FL1500 Imaging System (ThermoFisher, Rockford, IL) in using ChemiBlot SmartCapture.

.
