# Supplementary material for: Human α-galactosidase A is stimulated by folic acid supplementation – possible implications in Fabry disease management
Source: PLoS One. 2026 Jun 10;21(6):e0351438. doi: 10.1371/journal.pone.0351438 (PMC13252739; doi:10.1371/journal.pone.0351438)
Supplement: Attachment — Submitted filename: Response to Reviewers.docx [file pone.0351438.s006.docx]

Reviewer #1:

Comment: There is no …. should be mentioned.

Authors response: We thank and appreciate this valuable comment by the reviewer. The reviewer has also stated that since pure recombinant alpha-Gal A (AGLA) enzyme is also responding to folic acid (FA), the effect could be genuine. We nevertheless have repeated all cell lysate AGLA activity experiments in the presence of N-acetyl-D-galactosamine (NAG) and updated Figures 1 and 6 with new values determined. Methods section has been updated with new procedure (lines 182-185).

Comment: Authors also state that ….. supplementary figures for transparency.

Authors response: We thank this valuable comment by both reviewers (1 and 3). We have moved the Table from the manuscript to Supplementary Information (Supplementary Information S1 Table) that shows enzyme activities determined in presence of increasing FA concentrations with respective references.

Comment: Statistical analysis …..cannot be reviewed.

Authors response: We have mentioned the detailed statistical analysis in the revised manuscript (lines 203-206).

Comment: It should also ….. expressed isoform.

Authors response: The primer pair was purchased from commercial source, Origene. It amplifies a ~107 bp fragment about 150 bp into the ORF. It amplifies isoforms 1, 3, 4 and 5. In our amplifications, a single peak was displayed in melting curve. We have included this information in the Materials and Methods section of the revised manuscript (lines 157-161, 245-247).

Comment: Methylation analysis …. supplementary figure.

Authors response: We thank the reviewer for this suggestion. We have included methylation analysis in Supplementary Information (Supplementary Information S1 Fig and S2 Table).

Comment: The figure relative ….. been performed.

Authors response: We regret omission in our initial submission. We have included Figure 2C legend in the revised manuscript (lines 257-258 and 268-269). Our earlier work extensively analyzed several housekeeping gene's expression in relation to increasing folic acid concentrations. We repeatedly found that GAPDH expression is unchanged with folic acid concentrations tested in our earlier published studies, hence, included this widely used housekeeping protein for comparison.

Comment: It should be ….. of the protein.

Authors response: We thank the reviewer for this comment. We have made changes to discussion regarding the folic acid mediated stimulations at the transcript level and enzyme activity augmentation (lines 374-375).

Reviewer #2:

Comment: Even if the study …. classic phenotype.

Authors response: We agree with the reviewer's comment that the study needs evaluation in additional types of mutation in the *GLA* gene. We have acknowledged the limitation of study being used with a single cell line, in the discussion (lines 453-454). This study is an initial observation of folic acid stimulating the AGLA activity in cells and of recombinant pure enzyme. As a proof of concept, we also investigated whether FA improves deoxygalactonojirimycin (DGJ) mediated rescue in a patient's cell line.

Comment: Moreover, the results …. Increased AGAL level.

Authors response: We thank the reviewer for the suggestion and have converted the folic acid mediated increase of DGJ treated cell's activity to % of DGJ (lines 326-328).

Reviewer #3:

Authors response to comment 1: We thank the reviewer for valuable comment. We have included references regarding FD classification and modified the statement in the Introduction to include FD classification based on the reference (lines 54-59).

Authors response to comment 2: We have included aim and type of study in both abstract and introduction in the revised manuscript (lines 27-31 and 90-94).

Authors response to comment 3: We appreciate the observation by the reviewer. We have deleted the paragraph from the introduction in the revised manuscript.

Authors response to comment 4: We thank the reviewer for valuable feedback. Stock solution of sodium salt of folic acid was dissolved in water. It was diluted in either RPMI medium for cell exposure, or in citrate-phosphate buffer for recombinant enzyme assay. The maximum dilutions of folic acid we used in our study up to 500 ng/ml had no effect on fluorescence readout of 4MU. Fluorescent plate always had appropriate blanks; however, folic acid did not affect 4MU fluorescence. We have included these details in the revised manuscript material and methods section (lines 127-131).

Authors response to comment 5: We thank the reviewer for valuable feedback. We have included quantification methods (lines 157-161). Additionally, we have included additional information about the primer pair used (lines 245-247).

Authors response to comment 6: We thank the reviewer for valuable feedback. It was out inadvertent mistake of typing medians when we meant means. We have corrected the mistakes and included detailed statistical analysis statement in the revised manuscript (lines 203-206).

Authors response to comment 7: We appreciate this valuable comment by both reviewers (1 and 3). We have moved the Table from the manuscript to Supplementary Information S1 Table that shows enzyme activities determined in presence of increasing FA concentrations with references.

Authors response to comment 8: We thank the reviewer for this valuable feedback. Our study is limited by not measuring disease-relevant substrates, which we have acknowledged (lines 454-457). Our lab is not geared to make these measurements, perhaps in a future study we could collaborate with another group having ability towards measuring these substrates, which should conclusively show improvement in response to folic acid supplementation.

Authors response to comment 9: We thank the reviewer for valuable feedback. In the revised manuscript we have emphasized limitation of study to only one cell line for evaluating folic acid effect on deoxygalactonojirimycin rescue of AGLA activity (lines 453-454).

Authors response to comment 10: We have emphasized only one methylation region was interrogated in the current study. We are including supplementary information about the methylation sequence probed (Supplementary Information S1Fig and S2 Table).

Authors response to comment 11: We have clarified financial disclosures statement.

Authors response to comment 12: We appreciate the reviewer for pointing out quality of figures. In the revised manuscript, we have improved the figures according to journal guidelines.
