# Supplementary material for: Human α-galactosidase A is stimulated by folic acid supplementation – possible implications in Fabry disease management
Source: PLoS One. 2026 Jun 10;21(6):e0351438. doi: 10.1371/journal.pone.0351438 (PMC13252739; doi:10.1371/journal.pone.0351438)
Supplement: Attachment — Submitted filename: Response to Reviewer.docx [file pone.0351438.s007.docx]

**Reviewer #1**: I believe that ……. will be more accurate.

**Author Response**: We thank the reviewer for this valuable suggestion. We have performed ANOVA analyses as suggested and have incorporated statements in the manuscript. Because of ANOVA analyses, Figures 1, 2 and 6 were slightly modified to remove the asterisk and bar showing earlier t-test comparisons.
