# Supplementary material for: Human α-galactosidase A is stimulated by folic acid supplementation – possible implications in Fabry disease management
Source: PLoS One. 2026 Jun 10;21(6):e0351438. doi: 10.1371/journal.pone.0351438 (PMC13252739; doi:10.1371/journal.pone.0351438)
Supplement: Peer review history

# Peer review: Decision Letter 0

**DOI:** 10.1371/journal.pone.0351438.r001

**Authors:** Tatsuo Shimosawa

8 Feb 2026

PONE-D-25-67348

Human α-Galactosidase A is Stimulated by Folic Acid Supplementation – Possible Implications in Fabry Disease Management

PLOS One

Dear Dr. Junaid,

Thank you for submitting your manuscript to PLOS ONE. After careful consideration, we feel that it has merit but does not fully meet PLOS ONE’s publication criteria as it currently stands. Therefore, we invite you to submit a revised version of the manuscript that addresses the points raised during the review process.

The current study is interesting and contribute largely in inventing new treatment for Fabry Disease, however, the methods and results are not sufficient to support the conclusion.  Three experts raised several issues to be clarified.  Please revise extensively to draw the conclusion.

Please submit your revised manuscript by Mar 25 2026 11:59PM. If you will need more time than this to complete your revisions, please reply to this message or contact the journal office at plosone@plos.org. When you're ready to submit your revision, log on to https://www.editorialmanager.com/pone/ and select the 'Submissions Needing Revision' folder to locate your manuscript file.

Please include the following items when submitting your revised manuscript:

- A letter that responds to each point raised by the academic editor and reviewer(s). You should upload this letter as a separate file labeled 'Response to Reviewers'.
- A marked-up copy of your manuscript that highlights changes made to the original version. You should upload this as a separate file labeled 'Revised Manuscript with Track Changes'.
- An unmarked version of your revised paper without tracked changes. You should upload this as a separate file labeled 'Manuscript'.

If you would like to make changes to your financial disclosure, please include your updated statement in your cover letter. Guidelines for resubmitting your figure files are available below the reviewer comments at the end of this letter.

If applicable, we recommend that you deposit your laboratory protocols in protocols.io to enhance the reproducibility of your results. Protocols.io assigns your protocol its own identifier (DOI) so that it can be cited independently in the future. For instructions see: https://journals.plos.org/plosone/s/submission-guidelines#loc-laboratory-protocols. Additionally, PLOS ONE offers an option for publishing peer-reviewed Lab Protocol articles, which describe protocols hosted on protocols.io. Read more information on sharing protocols at https://plos.org/protocols?utm_medium=editorial-email&utm_source=authorletters&utm_campaign=protocols.

We look forward to receiving your revised manuscript.

Kind regards,

Tatsuo Shimosawa, M.D., Ph.D.

Academic Editor

PLOS One

Journal Requirements:

When submitting your revision, we need you to address these additional requirements.

1. Please ensure that your manuscript meets PLOS ONE's style requirements, including those for file naming. The PLOS ONE style templates can be found at

https://journals.plos.org/plosone/s/file?id=wjVg/PLOSOne_formatting_sample_main_body.pdf and

https://journals.plos.org/plosone/s/file?id=ba62/PLOSOne_formatting_sample_title_authors_affiliations.pdf

2. We note that you have included the phrase “data not shown” in your manuscript. Unfortunately, this does not meet our data sharing requirements. PLOS does not permit references to inaccessible data. We require that authors provide all relevant data within the paper, Supporting Information files, or in an acceptable, public repository. Please add a citation to support this phrase or upload the data that corresponds with these findings to a stable repository (such as Figshare or Dryad) and provide and URLs, DOIs, or accession numbers that may be used to access these data. Or, if the data are not a core part of the research being presented in your study, we ask that you remove the phrase that refers to these data.

3. Thank you for stating the following in the Acknowledgments Section of your manuscript:

“This study was supported by funds from the New York State Office for People with Developmental Disabilities.”

We note that you have provided additional information within the Acknowledgements Section that is not currently declared in your Funding Statement. Please note that funding information should not appear in the Acknowledgments section or other areas of your manuscript. We will only publish funding information present in the Funding Statement section of the online submission form.

Please remove any funding-related text from the manuscript and let us know how you would like to update your Funding Statement. Currently, your Funding Statement reads as follows:

“The author(s) received no specific funding for this work.”

Please include your amended statements within your cover letter; we will change the online submission form on your behalf.

4. We note that the grant information you provided in the ‘Funding Information’ and ‘Financial Disclosure’ sections do not match.

When you resubmit, please ensure that you provide the correct grant numbers for the awards you received for your study in the ‘Funding Information’ section

5. Thank you for stating the following financial disclosure:

“The author(s) received no specific funding for this work.”

At this time, please address the following queries:

a)        Please clarify the sources of funding (financial or material support) for your study. List the grants or organizations that supported your study, including funding received from your institution.

b)        State what role the funders took in the study. If the funders had no role in your study, please state: “The funders had no role in study design, data collection and analysis, decision to publish, or preparation of the manuscript.”

c)        If any authors received a salary from any of your funders, please state which authors and which funders.

d)        If you did not receive any funding for this study, please state: “The authors received no specific funding for this work.”

Please include your amended statements within your cover letter; we will change the online submission form on your behalf.

6. Please include your full ethics statement in the ‘Methods’ section of your manuscript file. In your statement, please include the full name of the IRB or ethics committee who approved or waived your study, as well as whether or not you obtained informed written or verbal consent. If consent was waived for your study, please include this information in your statement as well.

7. PLOS ONE now requires that authors provide the original uncropped and unadjusted images underlying all blot or gel results reported in a submission’s figures or Supporting Information files. This policy and the journal’s other requirements for blot/gel reporting and figure preparation are described in detail at https://journals.plos.org/plosone/s/figures#loc-blot-and-gel-reporting-requirements and https://journals.plos.org/plosone/s/figures#loc-preparing-figures-from-image-files. When you submit your revised manuscript, please ensure that your figures adhere fully to these guidelines and provide the original underlying images for all blot or gel data reported in your submission. See the following link for instructions on providing the original image data: https://journals.plos.org/plosone/s/figures#loc-original-images-for-blots-and-gels.

In your cover letter, please note whether your blot/gel image data are in Supporting Information or posted at a public data repository, provide the repository URL if relevant, and provide specific details as to which raw blot/gel images, if any, are not available. Email us at plosone@plos.org if you have any questions.

8. We note that your Data Availability Statement is currently as follows: “All relevant data are within the manuscript and its Supporting Information files.”

Please confirm at this time whether or not your submission contains all raw data required to replicate the results of your study. Authors must share the “minimal data set” for their submission. PLOS defines the minimal data set to consist of the data required to replicate all study findings reported in the article, as well as related metadata and methods (https://journals.plos.org/plosone/s/data-availability#loc-minimal-data-set-definition).

For example, authors should submit the following data:

- The values behind the means, standard deviations and other measures reported;

- The values used to build graphs;

- The points extracted from images for analysis.

Authors do not need to submit their entire data set if only a portion of the data was used in the reported study.

If your submission does not contain these data, please either upload them as Supporting Information files or deposit them to a stable, public repository and provide us with the relevant URLs, DOIs, or accession numbers. For a list of recommended repositories, please see https://journals.plos.org/plosone/s/recommended-repositories.

If there are ethical or legal restrictions on sharing a de-identified data set, please explain them in detail (e.g., data contain potentially sensitive information, data are owned by a third-party organization, etc.) and who has imposed them (e.g., an ethics committee). Please also provide contact information for a data access committee, ethics committee, or other institutional body to which data requests may be sent. If data are owned by a third party, please indicate how others may request data access.

9. If the reviewer comments include a recommendation to cite specific previously published works, please review and evaluate these publications to determine whether they are relevant and should be cited. There is no requirement to cite these works unless the editor has indicated otherwise.

[Note: HTML markup is below. Please do not edit.]

Reviewers' comments:

Reviewer's Responses to Questions

**Comments to the Author**

1. Is the manuscript technically sound, and do the data support the conclusions?

The manuscript must describe a technically sound piece of scientific research with data that supports the conclusions. Experiments must have been conducted rigorously, with appropriate controls, replication, and sample sizes. The conclusions must be drawn appropriately based on the data presented.

Reviewer #1: Partly

Reviewer #2: Yes

Reviewer #3: Partly

**********

2. Has the statistical analysis been performed appropriately and rigorously?

Reviewer #1: I Don't Know

Reviewer #2: I Don't Know

Reviewer #3: No

**********

3. Have the authors made all data underlying the findings in their manuscript fully available?

The PLOS Data policy requires authors to make all data underlying the findings described in their manuscript fully available without restriction, with rare exception (please refer to the Data Availability Statement in the manuscript PDF file). The data should be provided as part of the manuscript or its supporting information, or deposited to a public repository. For example, in addition to summary statistics, the data points behind means, medians and variance measures should be available. If there are restrictions on publicly sharing data—e.g. participant privacy or use of data from a third party—those must be specified.

Reviewer #1: No

Reviewer #2: Yes

Reviewer #3: No

**********

4. Is the manuscript presented in an intelligible fashion and written in standard English?

PLOS ONE does not copyedit accepted manuscripts, so the language in submitted articles must be clear, correct, and unambiguous. Any typographical or grammatical errors should be corrected at revision, so please note any specific errors here.

Reviewer #1: Yes

Reviewer #2: Yes

Reviewer #3: No

**********

5. Review Comments to the Author

Please use the space provided to explain your answers to the questions above. You may also include additional comments for the author, including concerns about dual publication, research ethics, or publication ethics. (Please upload your review as an attachment if it exceeds 20,000 characters)

Reviewer #1: The manuscript describes a possible interesting effect of folic acid on alpha-galactosidase A expression and activity, which could be helpful for the treatment of Fabry Disease.

Numerous experiments were performed to support the conclusions, however some of them are not methodologically precise or not enough described, therefore these aspects have to be clarified, emended or at least some methodological deficiency has to be mentioned as a limitation.

In particular:

There is no mention to the fact that N-Acetyl-galactosamine was added as a reagent in the activity tests performed in all cell lysates. This compound is used to exclude the contribution of the alpha-gal isoform B (or alpha-N-Acetylgalactosaminidase Ec 3.2.1.49 encoded by NAGA) to the enzymatic activity, since the two enzyme are homoulogus in their sequence and share the same substrate. Ideally, lysate activity assays should be repeated in the presence of N-Acetyl-galactosamine, however, since the experiments on the purified alpha-Gal A activity also show an effect of folic acid, it is assumable that the effect on the cell lysates could also be genuinely due to folic acid action on the correct isoform, but the limitation of the assays performed in cell lysate should be mentioned.

As a consequence, the sentence in page 10 line 205, in which author says that data obtained in lymphoblasts are "highly specific", should be avoided because the effect of the most important isoform of the enzyme was not excluded, according to the draft.

Authors also state that they have performed similar experiments in other lysosomal enzyme with no effect on folic acid. These experiments are not included in the manuscript, presumibly to simplify the reading and center the attention on the relevant results, but these experiments could be included as supplementary figures for transparency.

Statistical analysis was performed and it was indicated in the text which are the significant values, however not all the graphs include significant data representation and, more importantly, there is no mention in the whole manuscript on which tests were applied to analyze statistical significance, therefore statistical analysis accuracy cannot be reviewed.

It should also be clearly indicated in the text, which region of the enzyme or which exons are amplified in the m-RNA expression analysis and confirm that the primers are specific for GLA expressed isoform.

Methylation analysis of GLA could also be included as supplementary figure.

The figure relative to Western Blot data includes bands intensity quantification, but panel C is not mentioned in the caption. According to Western blot data, the quantification was performed on the bases of the relative expression of GLA compared to house keeping gene GAPDH, however it cannot be excluded that level of expression of GAPDH are also affected by folic acid, therefore quantification of additional house keeping genes or quantification of GLA relative to total protein load could have been performed.

It should be made more clear in the discussion that the presented experiments appoint to a double mechanism of folic acid to stimulate alpha-Galactosidase A production at the expression level and also determine a possible direct interaction with the protein which apparently does not involve the active site of the protein.

Other important limitations of the manuscript are correctly described by the authors (luck of in vivo effect, luck of data to support a clear mechanism such as the identification of the binding site for folic acid or the mechanism that support the increase in GLA m-RNA production).

Reviewer #2: I read with interest the article Human α-Galactosidase A is Stimulated by Folic Acid Supplementation – Possible Implications in Fabry Disease Management by Sabiha Khatoon.

The study reports the effect of a FA supplementation in augmenting AGLA enzymatic activity in-vivo and in a patients with Fabry disease.

Even if the study represents a news and an innovative trial to find further material for FD treatment , I believe that the sample results scarcely representative. The application of the FA supplementation in a single cell-line in a single patients (besides with a late-onset phenotype) represents an important limitation. It could be more interesting if the authors investigate also patients with missense mutation and classic phenotype.

Moreover, the results should better specify the exact percentage or n.of fold of increased AGAL level (compared to the 1.2 fold, 3% of DGJ).

Reviewer #3: General Comments

This study analyses the effect of folic acid supplementation on GLA/α-galactosidase A expression and enzymatic activity, and explores whether FA may enhance DGJ (migalastat)-mediated rescue in a Fabry disease lymphoblastoid cell line. Although the topic is innovative and potentially translationally relevant, several methodological issues should be addressed.

Specific revision comments

1) In Introduction (line 57–60), this paragraph is misleading; FD classification should follow clinical history, GLA gene analysis, α-Gal A assay, and blood accumulation of LysoGb3. (reference: Int. J. Mol. Sci. 2018, 19, 3726; doi:10.3390/ijms19123726). Please discuss this point, adding the reference.

2) In Introduction (also in Abstract), the aims and type of study should be clearly stated at the end of this section (e.g., experimental design, models used, and primary endpoints).

3) In Introduction (line 93–104), the paragraph summarizes the results of the study; therefore, it is out of context and should be erased or moved to the Results/Discussion section.

4) In Methods, AGLA activity is quantified by 4-MU fluorescence after stopping the reaction at pH 11. Since FA is a chromophore and can alter optical readouts and/or quench/enhance fluorescence depending on conditions and may also affect micro-pH or ionic strength, please provide more details regarding this issue.

5) In Methods, the term “Quantitative RT-PCR” should be better defined, specifying quantification method (e.g., ΔΔCt), amplification chemistry, primer efficiencies, reference gene strategy, and whether the plotted metric is truly “fold change” (including how normalization was performed).

6) In Statistics, this section is not sufficient. Authors state: “Each experiment was performed in triplicates. Values are presented as means ± SEM.” However, multiple figure captions say values are medians of three independent experiments with SEM bars. Additionally: Are the “triplicates” technical replicates within one culture, or independent biological replicates? Why are medians used with SEM ? What statistical tests were used, given multiple FA concentrations compared to baseline? Please clarify n, define what is meant by “independent experiments,” adopt consistent summary statistics, and report the full statistical workflow.

7) In Results, Authors state several lysosomal enzymes are unaffected, referring to Table 1. However, Table 1 is only a list of enzymes, not the quantitative activities, variability, or statistical comparisons. Please provide enzyme activity values (or normalized activities) across FA doses with statistics, at least for the most relevant comparator enzymes.

8) In Results, please report disease-relevant substrates, such as Gb3 and/or lyso-Gb3, to support biological relevance beyond enzyme activity.

9) In Discussion, only one FD lymphoblastoid line is tested (male with the Asn215Ser variant and mild manifestations). Considering the wide spectrum of FD, these results cannot be generalized to all FD populations. Please acknowledge this limitation, nuancing the discussion and clearly delimiting the scope of inference.

10) In Conclusion (also in Abstract), Authors state no methylation differences in one CpG island. However, this is insufficient to support conclusions about epigenetic mechanisms. Please nuance the conclusions based on the actual scope of methylation analysis (single region interrogated) and avoid broader mechanistic claims unless additional data are provided.

11) In Competing Interest Statement, Authors state “no competing interest to declare” but also state “Patent Applied For” with a USPTO application number. In addition, the submission form text indicates “no specific funding,” while the Acknowledgments say the study “was supported by funds” from a state office. Please verify disclosures.

12) Figures, the quality should be improved.

**********

6. PLOS authors have the option to publish the peer review history of their article (what does this mean?). If published, this will include your full peer review and any attached files.

If you choose “no”, your identity will remain anonymous but your review may still be made public.

**Do you want your identity to be public for this peer review?** For information about this choice, including consent withdrawal, please see our Privacy Policy.

Reviewer #1: **Yes:** Saida Ortolano

Reviewer #2: No

Reviewer #3: No

**********

[NOTE: If reviewer comments were submitted as an attachment file, they will be attached to this email and accessible via the submission site. Please log into your account, locate the manuscript record, and check for the action link "View Attachments". If this link does not appear, there are no attachment files.]

To ensure your figures meet our technical requirements, please review our figure guidelines: https://journals.plos.org/plosone/s/figures

You may also use PLOS’s free figure tool, NAAS, to help you prepare publication quality figures: https://journals.plos.org/plosone/s/figures#loc-tools-for-figure-preparation.

NAAS will assess whether your figures meet our technical requirements by comparing each figure against our figure specifications.

# Author comment: Author response to Decision Letter 1

**DOI:** 10.1371/journal.pone.0351438.r002

27 Mar 2026

Responses to all reviewer comments are present in the file "Responses to Reviewers".

Responses to all editorial comments are present in the file "Cover letter_Revision".

Attachment Submitted filename: Response to Reviewers.docx

# Peer review: Decision Letter 1

**DOI:** 10.1371/journal.pone.0351438.r003

**Authors:** Tatsuo Shimosawa

20 Apr 2026

PONE-D-25-67348R1

Human α-galactosidase A is stimulated by folic acid supplementation – possible implications in Fabry disease management

PLOS One

Dear Dr. Junaid,

Thank you for submitting your manuscript to PLOS ONE. After careful consideration, we feel that it has merit but does not fully meet PLOS ONE’s publication criteria as it currently stands. Therefore, we invite you to submit a revised version of the manuscript that addresses the points raised during the review process.

Please submit your revised manuscript by Jun 04 2026 11:59PM. If you will need more time than this to complete your revisions, please reply to this message or contact the journal office at plosone@plos.org. When you're ready to submit your revision, log on to https://www.editorialmanager.com/pone/ and select the 'Submissions Needing Revision' folder to locate your manuscript file.

Please include the following items when submitting your revised manuscript:

- A letter that responds to each point raised by the academic editor and reviewer(s). You should upload this letter as a separate file labeled 'Response to Reviewers'.
- A marked-up copy of your manuscript that highlights changes made to the original version. You should upload this as a separate file labeled 'Revised Manuscript with Track Changes'.
- An unmarked version of your revised paper without tracked changes. You should upload this as a separate file labeled 'Manuscript'.

If you would like to make changes to your financial disclosure, please include your updated statement in your cover letter. Guidelines for resubmitting your figure files are available below the reviewer comments at the end of this letter.

If applicable, we recommend that you deposit your laboratory protocols in protocols.io to enhance the reproducibility of your results. Protocols.io assigns your protocol its own identifier (DOI) so that it can be cited independently in the future. For instructions see: https://journals.plos.org/plosone/s/submission-guidelines#loc-laboratory-protocols. Additionally, PLOS ONE offers an option for publishing peer-reviewed Lab Protocol articles, which describe protocols hosted on protocols.io. Read more information on sharing protocols at https://plos.org/protocols?utm_medium=editorial-email&utm_source=authorletters&utm_campaign=protocols.

As the corresponding author, your ORCID iD is verified in the submission system and will appear in the published article. PLOS supports the use of ORCID, and we encourage all coauthors to register for an ORCID iD and use it as well. Please encourage your coauthors to verify their ORCID iD within the submission system before final acceptance, as unverified ORCID iDs will not appear in the published article. *Only* the individual author can complete the verification step; PLOS staff *cannot* verify ORCID iDs on behalf of authors.

We look forward to receiving your revised manuscript.

Kind regards,

Tatsuo Shimosawa, M.D., Ph.D.

Academic Editor

PLOS One

Journal Requirements:

If the reviewer comments include a recommendation to cite specific previously published works, please review and evaluate these publications to determine whether they are relevant and should be cited. There is no requirement to cite these works unless the editor has indicated otherwise.

Please review your reference list to ensure that it is complete and correct. If you have cited papers that have been retracted, please include the rationale for doing so in the manuscript text, or remove these references and replace them with relevant current references. Any changes to the reference list should be mentioned in the rebuttal letter that accompanies your revised manuscript. If you need to cite a retracted article, indicate the article’s retracted status in the References list and also include a citation and full reference for the retraction notice.

**Additional Editor Comments:**

Almost all the concerns were clarified, however, one expert has comments to be answered.

[Note: HTML markup is below. Please do not edit.]

Reviewers' comments:

Reviewer's Responses to Questions

**Comments to the Author**

1. If the authors have adequately addressed your comments raised in a previous round of review and you feel that this manuscript is now acceptable for publication, you may indicate that here to bypass the “Comments to the Author” section, enter your conflict of interest statement in the “Confidential to Editor” section, and submit your "Accept" recommendation.

Reviewer #1: (No Response)

Reviewer #2: All comments have been addressed

Reviewer #3: All comments have been addressed

**********

2. Is the manuscript technically sound, and do the data support the conclusions?

The manuscript must describe a technically sound piece of scientific research with data that supports the conclusions. Experiments must have been conducted rigorously, with appropriate controls, replication, and sample sizes. The conclusions must be drawn appropriately based on the data presented.

Reviewer #1: Partly

Reviewer #2: Yes

Reviewer #3: Yes

**********

3. Has the statistical analysis been performed appropriately and rigorously?

Reviewer #1: No

Reviewer #2: Yes

Reviewer #3: Yes

**********

4. Have the authors made all data underlying the findings in their manuscript fully available?

The PLOS Data policy requires authors to make all data underlying the findings described in their manuscript fully available without restriction, with rare exception (please refer to the Data Availability Statement in the manuscript PDF file). The data should be provided as part of the manuscript or its supporting information, or deposited to a public repository. For example, in addition to summary statistics, the data points behind means, medians and variance measures should be available. If there are restrictions on publicly sharing data—e.g. participant privacy or use of data from a third party—those must be specified.

Reviewer #1: Yes

Reviewer #2: Yes

Reviewer #3: No

**********

5. Is the manuscript presented in an intelligible fashion and written in standard English?

PLOS ONE does not copyedit accepted manuscripts, so the language in submitted articles must be clear, correct, and unambiguous. Any typographical or grammatical errors should be corrected at revision, so please note any specific errors here.

Reviewer #1: Yes

Reviewer #2: Yes

Reviewer #3: No

**********

6. Review Comments to the Author

Please use the space provided to explain your answers to the questions above. You may also include additional comments for the author, including concerns about dual publication, research ethics, or publication ethics. (Please upload your review as an attachment if it exceeds 20,000 characters)

Reviewer #1: I believe that the comments from the reviewers were well addressed and the manuscript improved considerably is quality, however in the statistical analysis author say that they have performed a student t-test, while the comparison of more than two conditions should be done by ANOVA, parametrics or non parametric test, depending on the case. This change won't change the general finding and will be more accurate.

Reviewer #2: the revised form of the article has reached almost of reviewer' suggestions and comments and is now suitable for publication

Reviewer #3: The authors have satisfactorily addressed all the concerns I had with the previous submission and made the necessary changes in the manuscript.

**********

7. PLOS authors have the option to publish the peer review history of their article (what does this mean?). If published, this will include your full peer review and any attached files.

If you choose “no”, your identity will remain anonymous but your review may still be made public.

**Do you want your identity to be public for this peer review?** For information about this choice, including consent withdrawal, please see our Privacy Policy.

Reviewer #1: **Yes:** Saida Ortolano

Reviewer #2: No

Reviewer #3: No

**********

[NOTE: If reviewer comments were submitted as an attachment file, they will be attached to this email and accessible via the submission site. Please log into your account, locate the manuscript record, and check for the action link "View Attachments". If this link does not appear, there are no attachment files.]

To ensure your figures meet our technical requirements, please review our figure guidelines: https://journals.plos.org/plosone/s/figures

You may also use PLOS’s free figure tool, NAAS, to help you prepare publication quality figures: https://journals.plos.org/plosone/s/figures#loc-tools-for-figure-preparation.

NAAS will assess whether your figures meet our technical requirements by comparing each figure against our figure specifications.

# Author comment: Author response to Decision Letter 2

**DOI:** 10.1371/journal.pone.0351438.r004

20 May 2026

We have performed ANOVA analyses as suggested by Reviewer 1, and have incorporated statements in the manuscript. As a result of this, Figures 1, 2 and 6 were slightly modified to remove asterisk and bar showing earlier t-test comparison. We have also included an acknowledgement statement to reflect help from Dr. Michael Flory, our Institute's Research Design and Analysis Services.

Attachment Submitted filename: Response to Reviewer.docx

# Peer review: Decision Letter 2

**DOI:** 10.1371/journal.pone.0351438.r005

**Authors:** Tatsuo Shimosawa

28 May 2026

Human α-galactosidase A is stimulated by folic acid supplementation – possible implications in Fabry disease management

PONE-D-25-67348R2

Dear Dr. Junaid,

We’re pleased to inform you that your manuscript has been judged scientifically suitable for publication and will be formally accepted for publication once it meets all outstanding technical requirements.

Within one week, you’ll receive an e-mail detailing the required amendments. When these have been addressed, you’ll receive a formal acceptance letter and your manuscript will be scheduled for publication.

An invoice will be generated when your article is formally accepted. Please note, if your institution has a publishing partnership with PLOS and your article meets the relevant criteria, all or part of your publication costs will be covered. Please make sure your user information is up-to-date by logging into Editorial Manager at Editorial Manager® and clicking the ‘Update My Information' link at the top of the page. For questions related to billing, please contact billing support.

If your institution or institutions have a press office, please notify them about your upcoming paper to help maximize its impact. If they’ll be preparing press materials, please inform our press team as soon as possible -- no later than 48 hours after receiving the formal acceptance. Your manuscript will remain under strict press embargo until 2 pm Eastern Time on the date of publication. For more information, please contact onepress@plos.org.

Kind regards,

Tatsuo Shimosawa, M.D., Ph.D.

Academic Editor

PLOS One

Additional Editor Comments (optional):

Reviewers' comments:

Reviewer's Responses to Questions

**Comments to the Author**

1. If the authors have adequately addressed your comments raised in a previous round of review and you feel that this manuscript is now acceptable for publication, you may indicate that here to bypass the “Comments to the Author” section, enter your conflict of interest statement in the “Confidential to Editor” section, and submit your "Accept" recommendation.

Reviewer #1: All comments have been addressed

**********

2. Is the manuscript technically sound, and do the data support the conclusions?

The manuscript must describe a technically sound piece of scientific research with data that supports the conclusions. Experiments must have been conducted rigorously, with appropriate controls, replication, and sample sizes. The conclusions must be drawn appropriately based on the data presented.

Reviewer #1: Yes

**********

3. Has the statistical analysis been performed appropriately and rigorously?

Reviewer #1: Yes

**********

4. Have the authors made all data underlying the findings in their manuscript fully available?

The PLOS Data policy requires authors to make all data underlying the findings described in their manuscript fully available without restriction, with rare exception (please refer to the Data Availability Statement in the manuscript PDF file). The data should be provided as part of the manuscript or its supporting information, or deposited to a public repository. For example, in addition to summary statistics, the data points behind means, medians and variance measures should be available. If there are restrictions on publicly sharing data—e.g. participant privacy or use of data from a third party—those must be specified.

Reviewer #1: Yes

**********

5. Is the manuscript presented in an intelligible fashion and written in standard English?

PLOS ONE does not copyedit accepted manuscripts, so the language in submitted articles must be clear, correct, and unambiguous. Any typographical or grammatical errors should be corrected at revision, so please note any specific errors here.

Reviewer #1: Yes

**********

6. Review Comments to the Author

Please use the space provided to explain your answers to the questions above. You may also include additional comments for the author, including concerns about dual publication, research ethics, or publication ethics. (Please upload your review as an attachment if it exceeds 20,000 characters)

Reviewer #1: Authors applied all the suggested improvements to the submitted article. The statistical analysis was correctly modified.

**********

7. PLOS authors have the option to publish the peer review history of their article (what does this mean?). If published, this will include your full peer review and any attached files.

If you choose “no”, your identity will remain anonymous but your review may still be made public.

**Do you want your identity to be public for this peer review?** For information about this choice, including consent withdrawal, please see our Privacy Policy.

Reviewer #1: **Yes:** Saida Ortolano

**********

# Editor report: Acceptance letter

**DOI:** 10.1371/journal.pone.0351438.r006

**Authors:** Tatsuo Shimosawa

PONE-D-25-67348R2

PLOS One

Dear Dr. Junaid,

I'm pleased to inform you that your manuscript has been deemed suitable for publication in PLOS One. Congratulations! Your manuscript is now being handed over to our production team.

At this stage, our production department will prepare your paper for publication. This includes ensuring the following:

* All references, tables, and figures are properly cited

* All relevant supporting information is included in the manuscript submission,

* There are no issues that prevent the paper from being properly typeset

You will receive further instructions from the production team, including instructions on how to review your proof when it is ready. Please keep in mind that we are working through a large volume of accepted articles, so please give us a few days to review your paper and let you know the next and final steps.

Lastly, if your institution or institutions have a press office, please let them know about your upcoming paper now to help maximize its impact. If they'll be preparing press materials, please inform our press team within the next 48 hours. Your manuscript will remain under strict press embargo until 2 pm Eastern Time on the date of publication. For more information, please contact onepress@plos.org.

You will receive an invoice from PLOS for your publication fee after your manuscript has reached the completed accept phase. If you receive an email requesting payment before acceptance or for any other service, this may be a phishing scheme. Learn how to identify phishing emails and protect your accounts at https://explore.plos.org/phishing.

If we can help with anything else, please email us at customercare@plos.org.

Thank you for submitting your work to PLOS ONE and supporting open access.

Kind regards,

PLOS ONE Editorial Office Staff

on behalf of

Prof. Tatsuo Shimosawa

Academic Editor

PLOS One
